# Thirty years after Alma-Ata: a systematic review of the impact of community health workers delivering curative interventions against malaria, pneumonia and diarrhoea on child mortality and morbidity in sub-Saharan Africa

**DOI:** 10.1186/1478-4491-9-27

**Published:** 2011-10-24

**Authors:** Jason B Christopher, Alex Le May, Simon Lewin, David A Ross

**Affiliations:** 1 PHDC Masters Programme, London School of Hygiene & Tropical Medicine, UK; 2 Norwegian Knowledge Centre for the Health Services, Norway and Medical Research Council of South Africa, South Africa; 3 Dept. of Infectious Disease Epidemiology, London School of Hygiene & Tropical Medicine, UK

## Abstract

**Background:**

Over thirty years have passed since the Alma-Ata Declaration on primary health care in 1978. Many governments in the first decade following the declaration responded by developing national programmes of community health workers (CHWs), but evaluations of these often demonstrated poor outcomes. As many CHW programmes have responded to the HIV/AIDS pandemic, international interest in them has returned and their role in the response to other diseases should be examined carefully so that lessons can be applied to their new roles. Over half of the deaths in African children under five years of age are due to malaria, diarrhoea and pneumonia - a situation which could be addressed through the use of cheap and effective interventions delivered by CHWs. However, to date there is very little evidence from randomised controlled trials of the impacts of CHW programmes on child mortality in Africa. Evidence from non-randomised controlled studies has not previously been reviewed systematically.

**Methods:**

We searched databases of published and unpublished studies for RCTs and non-randomised studies evaluating CHW programmes delivering curative treatments, with or without preventive components, for malaria, diarrhoea or pneumonia, in children in sub-Saharan Africa from 1987 to 2007. The impact of these programmes on morbidity or mortality in children under six years of age was reviewed. A descriptive analysis of interventional and contextual factors associated with these impacts was attempted.

**Results:**

The review identified seven studies evaluating CHWs, delivering a range of interventions. Limited descriptive data on programmes, contexts or process outcomes for these CHW programmes were available. CHWs in national programmes achieved large mortality reductions of 63% and 36% respectively, when insecticide-treated nets and anti-malarial chemoprophylaxis were delivered, in addition to curative interventions.

**Conclusions:**

CHW programmes could potentially achieve large gains in child survival in sub-Saharan Africa if these programmes were implemented at scale. Large-scale rigorous studies, including RCTs, are urgently needed to provide policymakers with more evidence on the effects of CHWs delivering these interventions.

## Background

In 1978, the Declaration of Alma-Ata presented Primary Health Care (PHC) as the means of achieving Health for All and community or lay health workers (CHWs) became a distinguishing feature of PHC implementation as it was rolled out. Several reviews of national CHW programmes in the late 1980s and early 1990s came to similar conclusions: quality of care from large-scale programmes was poor, generally because of a lack of ongoing training and supervision and poor logistical and financial support [1-3]. It has been argued that where national CHW programmes have failed, this has not been due to a failure of the concept of CHWs or PHC but because the support and supervision necessary to make them effective were too often missing. With the HIV/AIDS pandemic, and increasing acknowledgement of the critical shortage of human resources within health services to respond to it and to other diseases, the potential roles of CHWs within PHC have received renewed attention [4]. Recent developments confirm the growing recognition of the importance of PHC. It was the main subject of the 2008 WHO World Health Report, has the endorsement of WHO Director-General Margaret Chan [5], and was the topic of a themed issue of the Lancet [6].

Sub-Saharan Africa has only 3% of the global health workforce [7] but accounts for almost half of the 7.7 million child deaths globally [8,9]. 55% of these deaths in African children under 5 years of age are caused by malaria, pneumonia and diarrhoea [10]. Inexpensive interventions such as antibiotics, oral rehydration solution, insecticide-treated nets (ITNs) and antimalarials have been proven effective against these diseases, and it has been estimated that 65-91% of childhood deaths from these three diseases could be prevented if such interventions were delivered at scale in low-income countries [11]. Given the very limited professional health care human resources in these settings, it is important to examine the evidence for the effectiveness of CHW programmes as a delivery strategy for such interventions in sub-Saharan Africa. Whilst CHWs may deliver both preventive and curative interventions, this review focuses on the impact CHWs have when delivering curative interventions. The training and roles of CHWs who do not have any responsibility for the treatment of sick children are likely to be quite different from CHWs delivering curative interventions, and for this reason the review did not include the former.

Five recent reviews have examined CHW programmes. Lewin et al. [4] and Haines et al. [12], for example, conducted systematic and non-systematic reviews, respectively, that were broad in scope and were restricted to RCTs. The reviews identified only three assessments of CHWs' effectiveness from sub-Saharan Africa. A non-systematic review by Lehmann and Sanders [13] reported a broad range of evidence on CHWs. Beyond the three studies identified by Lewin et al. [4] they identified no further data on the impact of CHW programmes on morbidity/mortality in sub-Saharan Africa. Winch and colleagues [14] described the main models of CHW programmes addressing malaria and pneumonia in terms of drug delivery but did not assess their effectiveness. Finally, a recently published systematic review calculated a mortality impact estimate for interventions delivered by CHWs to preschool children [15]. However, only interventions against pneumonia were included in this research, and only one of the seven contributing studies was from Africa.

These reviews indicate that randomised controlled trial (RCT) evidence on the effectiveness of CHW programmes in sub-Saharan Africa is extremely scarce. While reviews have stressed the need for further health impact research, they have not considered the available evidence from non-randomised studies. This review attempts to rectify this by systematically reviewing randomised and non-randomised studies of CHWs' impact on child mortality in sub-Saharan Africa. The weaknesses of non-randomised studies have been described [16]. However, exclusion of all such studies without further consideration effectively places a zero weighting on evidence from non-randomised studies, which is clearly inappropriate. As long as the weaknesses of non-randomised studies are elucidated and taken into account, it is appropriate to evaluate the evidence from them, especially where RCTs are absent or few, in order to provide useful advice to policymakers on the impact of interventions[17,18].

This paper reports how we conducted the systematic review, an analysis of the studies identified by the review with descriptions of the CHW programmes they evaluated, and the observations and conclusions we have made.

## Methods

### Search strategy

Systematic reviews are summaries of research evidence that address a clearly formulated question using systematic and explicit methods to identify, select, and critically appraise relevant research, and to collect and analyse data from the studies that are included in the review [19]. For this systematic review, we searched the Medline (OVID), Embase (OVID) and CAB Direct databases, the last of which includes unpublished literature. CHW search terms from Lewin and colleagues' Cochrane review [4] were used, with permission. The search was limited to studies with a sub-Saharan African medical subject heading (MeSH) term, involving interventions directed, at least in part, at children less than six years of age, and that were delivered by CHWs who provided treatment (with or without preventive services) for malaria, diarrhoea or pneumonia. Searches were not limited by language. The disease-specific search filters were drawn from those used in the Medline (OVID) searches for the Cochrane systematic reviews of pneumonia [20], diarrhoea [21] and malaria [22]. The corresponding Embase MeSH terms and CAB Direct keyword searches were substituted for those used in the Medline(OVID) search. All the same limits and free-text searches were used (see Additional File 1).

All three database searches were developed iteratively. The titles and abstracts of the first 20 identified studies per database were analysed for additional relevant terms. Those not already within the respective database search were added to increase the search sensitivity. Titles and abstracts were studied for relevance. Full-text articles were located for those studies determined as potentially meeting the inclusion criteria. Two researchers with an interest in CHW programmes and child mortality were contacted to see if they knew of any additional unpublished or published data. We also examined the bibliographies of all full-text papers for further potential studies. Emails were sent to the authors of all included studies requesting details of any additional studies and to ask for further information on the characteristics of the CHW programme they had evaluated. Three landmark books on CHWs published between 1987 and 2007 were also examined for eligible studies [1-3].

### Definitions and inclusion criteria

#### Study Design

Randomised controlled trials (RCTs), controlled before and after (CBA), uncontrolled before and after, interrupted time series, and cohort and case control studies were included. Cross-sectional studies were excluded. We assessed risk of bias for included studies but did not exclude studies on this basis.

#### Study participants

A range of cadres with varied training and performing different roles have come under the umbrella term of CHW and it is thus difficult to provide a precise definition. For this review, we defined CHWs as individuals trained in the particular role of delivering curative care (with or without preventive health interventions) for malaria, pneumonia or diarrhoea to children aged less than six years. The intention was to evaluate CHWs who improve access to this curative care by working in community settings. However, in their liaison with other health workers, CHWs may spend some time in health centres. We did not want to exclude such CHWs from the review and therefore an additional criterion for inclusion was that CHWs worked, at least in part, outside medical facilities. Excluded from our definition were health workers who had received formal health training, apart from CHW training, and those who were formally accredited to a health worker cadre, such as nurses, paramedics or clinical officers. Teachers providing school-based activities would only have been included if they provided curative care to children aged less than six years. Mothers who had been trained to give anti-malarials from a pack to their own child were also excluded because they were not responsible for providing treatment outside their own families. Included studies were those that had evaluated the impact of interventions directed at children less than six years of age or where the impact in this age group (or part of this age group) was reported separately.

#### Interventions

We included CHWs delivering curative care, with or without preventive services, to children for at least one of malaria, pneumonia and diarrhoea. Programmes delivering purely preventive interventions (e.g. bed-net distribution and community-based hygiene education programmes) were excluded.

#### Effectiveness outcomes

Studies were included if they provided data on the impact of the CHW programme on mortality, morbidity or nutritional status in children under six years of age.

#### Region and time

We included studies conducted in sub-Saharan Africa between 1987 and 2007. This 20-year time-span was chosen because it covers the period following the three major earlier assessments of African CHW programmes [1-3]. We considered it unlikely that there were many eligible studies preceding this date and were concerned about changes in treatment delivery since the early 1980s.

Two reviewers (JC, AL) independently assessed all the titles and abstracts arising from the literature search for inclusion. A third reviewer (SL) was available as an independent arbiter when needed.

### Data extraction

Data extraction, and an assessment of risk of bias, was conducted independently by two reviewers using a common, pre-defined reporting matrix to summarise findings (see Additional file 2). Earlier evaluations of CHW programmes [1-3] identified important contextual and interventional determinants of effective CHW programmes (see 'Data Extraction: Characteristics which determine the effectiveness of CHW Programmes' section). Where possible this information was also extracted from study papers, references nd information obtained from the original authors.

### Assessment of risk of bias

Randomised Controlled Trials: RCTs were assessed with regard to attrition, performance and detection biases, concealment of allocation, use of intention-to-treat analyses and risk of contamination. Non-Randomised Studies: Data on potential confounders (see 'Data extracted on potential confounder' section) were extracted from articles, references and author-provided information in order to determine whether intervention and control groups were differentially affected. Risk of selection bias was assessed using the TREND checklist for the reporting of non-randomised studies [23].

### Data Extraction: Characteristics which determine the effectiveness of CHW Programmes

CONTEXTUAL FACTORS

Setting

Country, Rural/Urban

Healthcare setting: Home/Other

Context

Burden of malaria/diarrhoea/acute respiratory infection

Population characteristics (demography, sex, socio-economic status, cultural & religious background)

Functioning of basic health services

Decentralisation of health service control

Employment alternatives for CHWs

INTERVENTION FACTORS

CHW Programme Overview

Start date

Who set up & managed the programme(e.g. National Programme v NGO)

Number of CHWs in the programme

Number of total programme beneficiaries

Attrition rate of CHWs (i.e. how many of them stop being CHWs over time)

Paid or not, and if so by whom and how (e.g. cash or in kind)

CHW Roles

Curative & preventive health activities

Weekly pattern of activity

CHW Selection

By whom they were selected and the criteria used

CHW Characteristics

Education, sex, age, marital status, ethnicity, religion.

CHW Training

Duration, methods of training (e.g. didactic/practical), site (e.g. is it near their setting of work?), choice of trainers

Content of training (e.g. curative v preventive, record-keeping, training/education skills)

Refresher courses (how often, how long and by whom)

CHW Supervision

Who supervises? (eg villagers, PHC worker, government)

How do they supervise?

Existence of incentives for work of quality.

### Data extracted on potential confounders

➣ Alternative public/private health care provision

➣ NGO/mission healthcare provision

➣ Economic factors (e.g. improved economic status permitting better transport)

➣ Geographical factors (e.g. roads improving access to healthcare)

➣ Environmental factors (e.g. rains, famine)

### Analysis

Statistical pooling of outcome data was not attempted as the heterogeneity of the studies with regard to contextual and interventional factors would have rendered such a meta-analysis potentially misleading. Instead a narrative description of the results was conducted.

## Results

**Figure 1:**
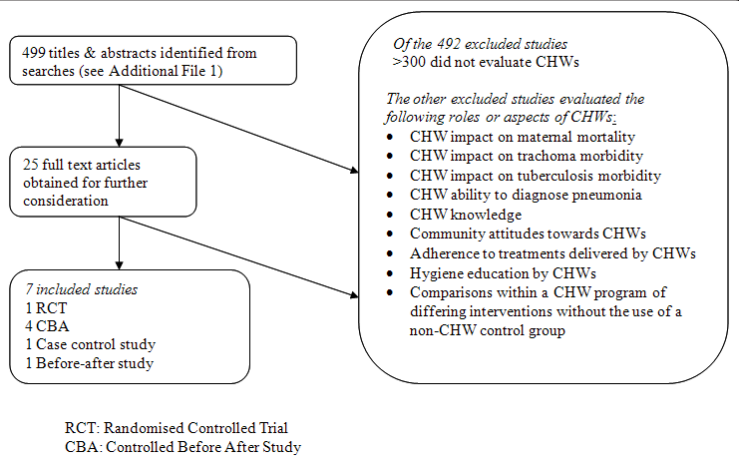
**Literature search chart**. CBA: Controlled before and after study.

The searches identified 499 unique titles and abstracts (see Figure 1). Screening of titles and abstracts revealed 25 titles that potentially met the inclusion criteria and full-text articles of these were obtained. Seven studies, published between 1991 and 2005, met the review's inclusion criteria.

### Description of included studies

The key characteristics of the studies are summarised in Table 1. For reporting here, each CHW programme has been given a short name, as outlined in Table 1.

**Table 1:** Characteristics and Findings of Studies

Study (first author, publication date, reference)	Setting	CHW programme	Intervention tested	Study design	Period when impact measured	Change in mortality (95% CIs)	Change in morbidity (95% CIs)
**Gambian PHC** **(Greenwood, 1988**, [26]**)**	North bank of river, Gambia. Rural	National programme (all villages > 400 people have a CHW)	13 CHWs delivering curative treatments, health education & malaria chemoprophylaxis	CBA	9-21 months after CHWs began delivering anti-malarial chemoprophylaxis	36% (-17, 63) reduction 1-4 yr old mortality	84% (48, 95) reduction in fever and parasitaemia

**Gambian PHC** **(Menon, 1990**, [24]**)**	North bank of river, Gambia. Rural	National programme (as above)	13 CHWs delivering curative treatments, health education & malaria chemoprophylaxis	CBA	3-4 yrs after CHWs began delivering anti-malarial chemoprophylaxis	77% (51, 89) reduction in 1-4 yr old mortality	84% (60, 94) reduction in fever and parasitaemia

**Gambian PHC** **(Hill, 2000**, [25]**)**	North bank of River Gambia. Rural	National programme (as above)	1 CHW & TBA per village (15 villages) Curative treatments & health education delivered.	CBA	Mortality measured in four successive 2-3 yr periods after programme onset in 1983, covering 14 years.	33% (10, 50) reduction in 1-4 yr old mortality, 6 to 9 yrs after programme onset.	Not assessed

**Gambian PHC** **(Alonso, 1991**, [27]**)**	South bank of river, Gambia. Rural	National programme (as above)	1 CHW & TBA per village (17 villages) delivering ITNs, curative treatments & health education	CBA	0-12 months following initiation of ITN delivery by CHWs	63% (32, 80) reduction in 1-4 yr old mortality	Not assessed

**Navrongo** **(Pence, 2005**, [28]**)**	North Ghana. Rural	Initiated by research institute	CHWs delivering health education, curative treatments, making referrals	Cluster RCT Only 4 clusters	4-5 years after CHWs rolled out	87% (27, 178) increase in 1-2 yr old mortality	Not assessed

**Gomoa** **(Afari, 1995**, [29]**)**	South Ghana. Rural	Initiated by research institute	Curative treatments & growth monitoring by 6 CHWs, 1 nurse & 1 physician.	Before and after study	0-3 years after programme onset.	61% (no CIs given) reduction in 0-4 yr old mortality, 36 months after programme onset	Not assessed

**Pahou** **(Velema, 1991**, [30]**)**	Coast of Benin. Rural	National programme	17 CHWs. Tasks included home visits, curative treatments, anti-malarial chemoprophylaxis, health education, growth monitoring, and referrals.	Case control study	Cases (deaths) & controls were assessed for exposure to CHWs in the preceding 3-year period	OR = 0.39 (0.16, 0.97)	Not assessed

CHW: Community Health Worker; RCT: Randomized Controlled Trial

ITN: Insecticide-treated nets; NGO: Non-governmental organisation

TBA: Traditional birth attendant; CBA: Controlled before and after study

OR: Odds ratio of death in children exposed to CHW compared to those unexposed

### Study settings

All seven CHW programmes were conducted in West Africa. Four studies evaluated CHWs in the Gambia [24-27]. Three of these studies [24-26] tested CHW programmes in the population of Farafenni, North Division. Menon and colleagues [24] and Greenwood and colleagues [26] studied CHWs delivering identical services in the same population and differing only with regard to when impact was assessed. Two studies assessed CHW programmes in Ghana [28,29] and one evaluated CHWs in Benin [30]. All studies were located in rural settings with high mortality and morbidity from diarrhoea, pneumonia and malaria.

### Study designs and outcomes

One study conducted in Ghana was an RCT [28] and four others were CBA studies, all based in the Gambia [24-27]. Also included were one uncontrolled before and after comparison [29] and one case-control study [30]. All studies measured impacts on infant and child mortality through the use of demographic surveillance systems. Malarial morbidity was also assessed in the Gambian studies.

### Characteristics of CHW programmes

The Gambian PHC programme and the Pahou programme in Benin were nationwide CHW interventions from which a selected group of CHWs were studied [27,30]. The Navrongo and Gomoa studies in Ghana were of small-scale CHW programmes initiated by research institutes at the time of the study [28,29]. The number of CHWs included in the studies ranged from 8 to 17. In the Gambian PHC and Navrongo projects, the CHWs were older men selected by village health committees [27,28]. The sex and selection of CHWs in the Gomoa and Pahou (Benin) projects were not reported.

Apart from the Gomoa project which did not report this information [29], all CHW programmes delivered health education on childhood nutrition, hygiene and immunisations, oral rehydration solution and dispensed chloroquine as anti-malarial chemotherapy as well as other unspecified medicines. Some CHW programmes provided paracetamol, mebendazole and multivitamins as well as growth monitoring. Pahou CHWs made referrals of patients to community health centres [30] but no mention was made of such a role in the other programmes. Gambian PHC was the only programme in which CHWs provided antibiotics (Penicillin V injections). Of the Gambian PHC programmes, Menon and colleagues [24] and Greenwood and colleagues [26] studied CHWs delivering maloprim for malarial chemoprophylaxis whilst Alonso and colleagues [27] studied CHWs delivering insecticide-treated nets (ITNs).

Navrongo CHWs were trained for 6 weeks [28] and Gambian PHC CHWs for 8 weeks [27]. The duration of training was not reported for the Pahou and Gomoa programmes. Little information was provided about the nature of this training or on the availability of further education after the initial course. CHW payment was noted to be informal and left to the discretion of the villagers in the Gambian PHC programme, and unreported for the other programmes, except for Navrongo where CHW work was clearly stated to be unpaid [28]. CHWs were supervised by a range of professionals, including community health nurses in Pahou and Gambian PHC [27,30], physicians and nurses in Gomoa [29] and by a village health committee in the Navrongo programme [28]. The services available to the comparison groups in the studies were poorly described. The Gambian national PHC programme only placed CHWs in villages with over 400 residents, and smaller villages were used as controls. Mobile teams delivering the Expanded Programme on Immunisation visited both the larger and smaller villages. The control group in the Navrongo study [28] received rural healthcare according to Ministry of Health guidelines. The other studies gave no descriptions of services for comparison groups.

### Risk of bias

The Navrongo study [28], using a cluster RCT design, randomised each of 4 clusters to receive a different health care delivery strategy, one of which was a control. Comparability of the clusters was compromised by differing baseline child mortality rates, and the fact that there was only one cluster in each study arm will have negated many of the potential advantages of randomisation [31]. The intervention was scaled up incrementally within each cluster. A comparison was made of the mortality rates between geographical areas where the intervention had and had not been scaled up. However, the process by which geographical areas within a cluster were chosen for initial scale up was not described and therefore the comparison of these areas may have been affected by selection bias.

Hill and colleagues [25], in reporting the findings of their CBA study in the Gambia, noted that better roads were built near the villages in the intervention group (PHC villages). This co-intervention, which took place during the study period, may have confounded the study findings since improved access to facility care for PHC villages relative to non-PHC (control) villages may have been responsible for the reduction in mortality observed following the intervention. No mortality reduction was evident before the roads were built [25].

The large impact on child mortality observed in the Gambian study by Menon and colleagues [24], which used a CBA design, was partly confounded by secular changes. Adjustment to determine if any residual impact might have been significant was not performed. For Greenwood and colleagues' CBA study in the Gambia [26], the confounders were a worse malaria season in the post-intervention measurement period and increased treatment from dispensaries in the control group. Since both of these worked to reduce the observed impact, the true reduction in mortality is likely to have been greater than the 36% reported (Table 1).

Alonso and colleagues' Gambian CBA study [27] evaluated CHWs whose main role during the study was ITN delivery. They considered the potential effects of a number of possible confounders. Confounding by differential access to anti-malarial chemotherapy between the intervention and control groups was excluded convincingly by the use of urinary chloroquine assessments. The authors also note the possibility that differences in village sizes and other factors may also have acted as confounders. However, they argue that the large 1-4 year old mortality reductions seen in the intervention sites, and the clear attribution of these reductions specifically to lower malaria mortality, makes the introduction of ITNs delivered by CHWs the most plausible explanation.

In their case control study in Benin, Velema and colleagues [30] assessed known potential confounders and selection biases (socioeconomic status, age, sex and the village from which the children came) and demonstrated that the measured impact was unlikely to have been due to them. However since this was a case-control study, unknown confounders and selection biases may have been responsible for the reduced likelihood of death in those receiving the CHW intervention. The before and after study by Afari and colleagues in Ghana [29] did not include a control group and made no attempt to identify and measure other potential explanations for the effects seen.

### Impacts

Four studies assessed mortality impact over 12 months. In addition, the Gomoa study in Ghana [29] measured mortality over 3 years, Hill and colleagues' study in the Gambia [25] measured mortality in 2-3 year time periods for a total of 14 years, and the Pahou study in Benin [30] used deaths over a 2 year period as cases in a case-control trial. The studies demonstrated varying impacts of CHW programmes on child mortality, ranging from a 63% reduction [27] to a 87% increase [28], with six out of seven studies showing a reduction overall, compared either with contemporaneous controls or in 'after' versus 'before' comparisons (Table 1).

It was a national programme of CHWs and traditional birth attendants (TBAs) delivering basic treatments, ITNs and health education in the Gambia which achieved a 63% reduction in mortality among 1-4 year olds [27]. When the same CHW programme delivered anti-malarial chemoprophylaxis (instead of ITNs), 1-4 year old mortality was reduced by 36% and the prevalence of children with fever and parasitaemia was reduced by 84% [26]. The impacts reported in the other five studies were less certain because of the biases described above. It was Pence and colleagues [28], who reported that a research-instituted CHW programme in Navrongo, Ghana was associated with a marked increase in 1-4 year old mortality within 4-5 years of its inception.

Apart from malarial morbidity, only one study (Gomoa) reported other measures of morbidity, measuring nutritional status in the before and after groups. The study found no statistically significant changes in height-for-age, or weight-for-height in children following the intervention.

Contamination as a result of children from control groups receiving care from nearby villages with CHWs was mentioned only in one study [25]. However, this may have occurred in all studies, apart from the Pahou study in Benin [30], thereby reducing the observed impact relative to true impact. The confidence intervals around the reported effect sizes for the studies were likely to be substantial underestimates since clustering was not adjusted for in any of the studies, even in those where there was only one cluster per study arm.

## Discussion

A recent overview of systematic reviews suggested that there is very little evidence on the effectiveness of different policy options for human resources, including the use of CHWs, in low-income countries [32]. Similarly, our review identified few studies published in the last 20 years on the impacts on child mortality and morbidity by sub-Saharan CHW programmes designed to deliver curative interventions against malaria, diarrhoea or pneumonia. However, several of the studies that were included had not been identified by the two global CHW reviews that included non-randomised designs [13,15]. This review therefore contributes towards developing the evidence base on the effects of CHW programmes. It does however also reveal that there may not be a large pool of non-randomised studies to draw upon when investigating the impact of CHW programmes on child health in Africa. Many reports of such programmes that were identified in our literature search did not include any evaluation of effectiveness against either mortality or morbidity.

It is unclear whether the finding that there is only a small pool of non-randomised studies conducted in Africa is generalizable to other regions. A recent Cochrane review of randomized controlled trials of CHW programmes identified nine trials from Asia and four from Africa, but did not consider non-randomized studies [4]. Further work is needed to explore the amount and quality of evidence from other high mortality regions.

The Gambian studies provide evidence that in a rural African setting affected by seasonal malaria, a national CHW programme delivering either ITNs or malarial chemoprophylaxis can have a marked impact on child mortality. This finding has important implications for child health care in settings in which professional providers are in short supply. The CHWs involved in the Gambian studies were selected by the villagers, were supervised by community nurses and paid minimally, if at all. Such a result is surprising given that it is smaller programmes with NGO or research institute involvement which have been typically associated with better outcomes [33]. These programmes are generally able to place greater emphasis on training, supervision, support and payment in cash or kind.

It is unclear whether the impacts reported, which were generally measured within two years of the initiation of the CHW intervention, would be sustained over longer periods. National programmes are often associated with high rates of CHW attrition [34,35] and initial enthusiasm may be undermined by the preference consumers often have for curative over preventive interventions. The Gambian study by Hill and colleagues [25], which had a considerably longer follow-up period of 14 years, showed that the 33% reduction in child mortality all occurred during the initial period of greatest investment in CHWs. After this period there was a decline in political and financial support for the programme, and no significant impact was measured subsequently. Although it is plausible that the CHWs were responsible for the mortality reduction, attribution would have been strengthened if potential confounders, such as improved access to health services through the construction of roads, had been studied and adjusted for.

CHW programmes can only be effective insofar as they deliver effective interventions. Only three studies provided any evaluation of which particular treatments delivered by the CHWs were responsible for the measured effects. In two of the included studies, the CHW interventions were randomised to include, or not include, malarial chemoprophylaxis [24,26] while one study randomised the delivery of ITNs by CHWs [27]. The results demonstrated that it was these preventive interventions as delivered by the CHWs, and not the other activities of the CHWs alone, which reduced childhood mortality. ITNs and malarial chemoprophylaxis are high-efficacy interventions and it is possible other interventions, such as health education, may be delivered equally well but without demonstrable impact. However, other attributes of the CHWs, such as their standing in the community, may have contributed to their effectiveness when they delivered ITNs and malarial chemoprophylaxis. This review was limited in scope to CHW programmes delivering curative interventions, with or without preventive ones, since it was concerned with the effectiveness of CHWs in improving access to health care rather than with their role in primary prevention. However these findings suggest that policymakers should prioritise these and other highly efficacious preventive interventions for application in CHW programmes.

Generalisation of this review's findings across Africa is problematic, since all seven studies occurred in West Africa and four were from The Gambia, with three having the same study population. These Gambian studies were undertaken in the 1980s, when government spending on primary health care exceeded that of hospital care for the first time in the Gambia [25]. Whilst villages were expected to generate payment for their CHWs, the nurses who supervised, educated and supplied CHWs were centrally funded for this role. CHW programmes in settings where there is less political will and financial investment may not have the necessary support for effective implementation and may therefore not achieve the impacts observed in these Gambian studies.

Further, the Gambian studies occurred in rural settings affected by seasonal malaria where a short period of good adherence to ITNs or chemoprophylaxis may result in larger mortality reductions than in settings where the malaria is less seasonal (such as Nigeria, Gabon and the Congo) and where good adherence needs to be maintained throughout the year. Settings where factors such as parasites' drug sensitivities, mosquito biting habits and the acceptability of ITNs and chemoprophylaxis differ from that in The Gambia are likely to result in differing impacts from similar CHW interventions.

An additional factor in the Gambian studies was the involvement of the Gambian Medical Research Council, which may have improved access to drugs and equipment or adherence to interventions in the study areas.

The studies selected by this review included little assessment of intermediate or process outcomes such as changes in health beliefs, increased use of primary care facilities or community empowerment. The failure of many evaluations of complex interventions to consider process issues adequately has been shown to limit the ability of investigators to account for the effects (or lack of effects) of interventions [36,37]. For example, in Greenwood and colleagues' [26] study in the Gambia, deaths in the PHC group were followed up and it was found that CHWs were frequently unavailable to children during the early stages of a febrile illness. This was because CHWs often found it necessary to work in the fields as their income from their role as a CHW was minimal. It would have been interesting to have had data on whether those who survived had better access to CHWs, but this was not reported.

Most studies did not report any unintended or adverse effects. However, the Navrongo study [28] observed an increase in mortality, principally in the 12 to 23-month age group. They speculated that mothers of children with diarrhoea and respiratory infections may have sought advice and basic treatment from CHWs thereby delaying or preventing the delivery of better treatment by more skilled providers in sub-district clinics. It is not possible to assess the validity of this hypothesis since no empirical evidence for or against it is presented. However this highlights the importance of considering and assessing potential unintended effects in evaluations of CHW programmes.

The possible impacts of the interventions on equity were not specifically addressed in the seven studies, although Hill and colleagues [25] found no significant differences in the impacts of CHWs on the three local ethnic groups in their Gambian study. An earlier systematic review of CHW interventions also found that impacts on equity were rarely considered [4]. However, in all study settings, dispensaries and health centres were only available in towns and large villages and were relatively inaccessible to rural villagers, so overall inequities in access to primary medical care by geographic and socio-economic status were likely to have been reduced by these village-based CHW interventions. The question of how CHW programmes can be linked to other components of a health system such as primary care facilities, health centres and private health care providers was also not addressed by the studies included in our review.

This review has several potential limitations. Firstly, it is possible that some published and unpublished CHW evaluations were not identified through the search strategies used. However, considerable effort was made to identify additional studies through contacting the authors of included studies and scanning the reference lists of existing books and papers. Secondly, our focus on studies from Africa may limit the generalizability of the review findings to other regions. Thirdly, our definition of CHWs may have excluded some cadres that others would consider to be lay health workers.

### Implications for research

Malaria, diarrhoea and pneumonia are of huge public health importance in sub-Saharan Africa and if ITNs, antimalarials, antibiotics, oral rehydration solution and other simple interventions were to be delivered at scale, millions of childhood deaths could be prevented annually. CHW programmes represent an important policy option for delivering these interventions in settings with limited human resources for health services and yet this review reveals such programmes continue to be neglected as a research priority.

The finding in this review of additional evidence suggesting that CHWs delivering antimalarial interventions, including preventive interventions only, can have a marked impact highlights further the urgent need for rigorous studies of the effects of these programmes on child mortality and morbidity. Once such primary studies have been conducted, it may be useful to conduct another review of the effects of CHWs delivering preventive interventions only.

Valid and reliable measurement of mortality is best obtained within a continuous demographic surveillance system which reports births, deaths, and out- and in- migration over a number of years [38]. With at least 23 such systems in sub-Saharan Africa now participating in the INDEPTH network [39], the potential for conducting community-based studies of the impacts of CHWs in Africa is more significant than ever before. Such studies should assess intermediate and process outcomes not only to explain measured impacts, but because such information helps policymakers determine whether the programme and impact can be replicated in other settings. In this review, very little information on CHW programme design and implementation was found (see 'Data Extraction: Characteristics which determine the effectiveness of CHW Programmes' section). Presentation of such information is also necessary for policy makers to consider the applicability of findings to different settings and in order to be able to replicate the interventions that were evaluated [40].

Although few studies were eligible for inclusion in this review, the reported mortality reductions were substantial for programmes in which CHWs were responsible for delivering ITNs or anti-malarial chemoprophylaxis [24,26,27]. Given the substantial improvement in child survival from these two interventions when successfully delivered, cluster randomised trials comparing the cost-effectiveness of delivery strategies involving CHWs compared with alternative strategies are indicated. Where RCTs are not possible, CBA studies with several years of observation and thorough documentation of likely confounders and process indicators should be conducted [38] and can provide strong plausibility inferences [41]. Stepped-wedge designs [42], should be considered in the evaluation of planned programmes as they take advantage of the typical incremental implementation of programmes across sites.

The most informative of the studies included in this review focussed on CHW interventions against malaria, a disease which is thought to account for 18% of under 5 year old mortality in Africa [10]. More research is needed on CHW impacts on pneumonia and diarrhoea, which are estimated to be responsible for 21% and 16% of child mortality in Africa, respectively [10]. Although a meta-analysis of pneumonia case management found community-based management achieved a mortality reduction of 27% [43], only one of the eleven studies included was from Africa. This particular study [44] was excluded from this review because it took place over 20 years ago.

Policymakers considering whether to implement CHW programmes need to consider other factors in addition to the evidence on impact. Such factors include programme feasibility (including costs), the low risk of adverse outcomes, acceptability, the potential for a large effect, and other beneficial health and social outcomes. All of these have been described as factors that would lower the threshold for the strength of the evidence needed before the recommendation of a public health intervention [17]. Feasibility, acceptability and other beneficial health and social outcomes will vary by context and local evidence is therefore needed to inform recommendations regarding CHW implementation in any one setting [45].

## Conclusion

Evidence from this review suggests that CHW programmes can have large impacts on child mortality when these programmes deliver ITNs or malarial chemoprophylaxis in an endemic malaria setting. Such reductions in mortality would bring about large gains in child survival in sub-Saharan Africa if these programmes were implemented at scale. However, 30 years after Alma-Ata there is still little evidence from Africa on the effectiveness of CHWs delivering curative interventions against pneumonia and diarrhoea or comprehensive packages of interventions against the major causes of mortality in children (pneumonia, diarrhoea, malaria, and, in some settings, HIV). Large-scale rigorous studies, including RCTs, are now urgently needed to provide policy makers with more evidence on the effectiveness of CHW programmes on child mortality.

## Competing interests

The authors declare that they have no competing interests.

## Authors' contributions

JC conceived the research topic and formulated the methods with advice from DR and SL. The data were extracted by JC and AL. JC wrote the first draft of the paper, and all authors contributed to the analysis and interpretation of the data and reviewed and edited the manuscript for important intellectual content. The opinions expressed are those of the authors alone. All authors read and approved the final manuscript.

## Supplementary Material

Additional file 1 **Database searches**. Click here for file

Additional file 2 Data extraction sheet Click here for file
