# Supplementary material for: Thirty years after Alma-Ata: a systematic review of the impact of community health workers delivering curative interventions against malaria, pneumonia and diarrhoea on child mortality and morbidity in sub-Saharan Africa
Source: Hum Resour Health. 2011 Oct 24;9:27. doi: 10.1186/1478-4491-9-27 (PMC3214180; doi:10.1186/1478-4491-9-27)
Supplement: Additional file 1 — Database searches. [file 1478-4491-9-27-S1.DOCX]

## Additional file 1 - Database searches

**Medline(OVID) Search**

1. Community Health Aides/

2. Home Health Aides/

3. Voluntary Workers/

4. Home Nursing/

5. Community Networks/

6. Peer Group/

7. Caregivers/

8. Social Support/

9. ((lay or voluntary or volunteer? or untrained or unlicensed or nonprofessional? or non professional?) adj5 (worker? or visitor? or attendant? or aid or aides or support$ or personnel or helper? or carer? or caregiver? or care giver? or consultant? or assistant? or staff or visit$ or midwife or midwives)).mp. [mp=title, original title, abstract, name of substance word, subject heading word]

10. lay volunteer?.mp.

11. paraprofessional?.mp. [mp=title, original title, abstract, name of substance word, subject heading word]

12. (paramedical adj (person$ or staff or aid or aides or assistant?)).mp. [mp=title, original title, abstract, name of substance word, subject heading word]

13. (trained adj3 (volunteer? or lay person$ or health worker? or mother?)).mp. [mp=title, original title, abstract, name of substance word, subject heading word]

14. ((community or primary or village?) adj3 (health worker? or health care worker? or healthcare worker?)).mp. [mp=title, original title, abstract, name of substance word, subject heading word]

15. (community adj3 (volunteer? or aid or aides or support)).mp. [mp=title, original title, abstract, name of substance word, subject heading word]

16. ((birth or childbirth or child birth or labor or labour) adj (attendant? or assistant?)).mp. [mp=title, original title, abstract, name of substance word, subject heading word]

17. (doula? or douladural?).mp. [mp=title, original title, abstract, name of substance word, subject heading word]

18. monitrice?.mp. [mp=title, original title, abstract, name of substance word, subject heading word]

19. (peer adj (volunteer? or counsel$ or outreach or support)).mp. [mp=title, original title, abstract, name of substance word, subject heading word]

20. "peer to peer".mp. [mp=title, original title, abstract, name of substance word, subject heading word]

21. "mother to mother".mp. [mp=title, original title, abstract, name of substance word, subject heading word]

22. "family to family".mp. [mp=title, original title, abstract, name of substance word, subject heading word]

23. (church based adj3 (intervention$ or program$ or counsel$)).mp. [mp=title, original title, abstract, name of substance word, subject heading word]

24. (linkworker? or link worker?).mp. [mp=title, original title, abstract, name of substance word, subject heading word]

25. barefoot doctor?.mp. [mp=title, original title, abstract, name of substance word, subject heading word]

26. (home adj (care or aid or aides or nursing or support or intervention? or treatment? or visit$)).mp. [mp=title, original title, abstract, name of substance word, subject heading word]

27. ((care or aid or aides or nursing or support or intervention? or treatment? or visit$) adj3 (lay or volunteer? or voluntary)).mp. [mp=title, original title, abstract, name of substance word, subject heading word]

28. 26 and 27

29. 1 or 2 or 3 or 4 or 5 or 6 or 7 or 8 or 9 or 10 or 11 or 12 or 13 or 14 or 15 or 16 or 17 or 18 or 19 or 20 or 21 or 22 or 23 or 24 or 25 or 28

30. Lay Health Worker.mp. [mp=title, original title, abstract, name of substance word, subject heading word]

31. "Letter [Publication Type]"/

32. "Editorial [Publication Type]"/

33. 29 or 30

34. 31 or 32

35. 33 not 34

36. acute lower respiratory infection.mp.

37. acute respiratory infection.mp.

38. exp Pneumonia/

39. pneumonia.mp. [mp=title, original title, abstract, name of substance word, subject heading word]

40. exp Anti-Bacterial Agents/

41. antibiotic$.mp. [mp=title, original title, abstract, name of substance word, subject heading word]

42. exp Child/

43. exp Infant/

44. (children or infant$ or pediatric or paediatric).mp. [mp=title, original title, abstract, name of substance word, subject heading word]

45. or/42-44

46. 36 or 37 or 38 or 39 or 40 or 41

47. malaria.mp. or exp Malaria/

48. exp Diarrhea/ or diarrhoea.mp.

49. Subsaharan Africa.mp. or exp "Africa South of the Sahara"/

50. Sub-saharan Africa.mp. [mp=title, original title, abstract, name of substance word, subject heading word]

51. 49 or 50

52. 46 or 47 or 48

53. 35 and 45 and 51 and 52

54. limit 53 to yr="1987 - 2007

**Embase (OVID)**

1. ((lay or voluntary or volunteer? or untrained or unlicensed or nonprofessional? or non professional?) adj5 (worker? or visitor? or attendant? or aid or aides or support$ or personnel or helper? or carer? or caregiver? or care giver? or consultant? or assistant? or staff or visit$ or midwife or midwives)).mp. [mp=title, abstract, subject headings, heading word, drug trade name, original title, device manufacturer, drug manufacturer name]

2. lay volunteer?.mp.

3. paraprofessional?.mp. [mp=title, abstract, subject headings, heading word, drug trade name, original title, device manufacturer, drug manufacturer name]

4. (paramedical adj (person$ or staff or aid or aides or assistant?)).mp. [mp=title, abstract, subject headings, heading word, drug trade name, original title, device manufacturer, drug manufacturer name]

5. (trained adj3 (volunteer? or lay person$ or health worker? or mother?)).mp. [mp=title, abstract, subject headings, heading word, drug trade name, original title, device manufacturer, drug manufacturer name]

6. ((community or primary or village?) adj3 (health worker? or health care worker? or healthcare worker?)).mp. [mp=title, abstract, subject headings, heading word, drug trade name, original title, device manufacturer, drug manufacturer name]

7. (community adj3 (volunteer? or aid or aides or support)).mp. [mp=title, abstract, subject headings, heading word, drug trade name, original title, device manufacturer, drug manufacturer name]

8. ((birth or childbirth or child birth or labor or labour) adj (attendant? or assistant?)).mp. [mp=title, abstract, subject headings, heading word, drug trade name, original title, device manufacturer, drug manufacturer name]

9. (doula? or douladural?).mp. [mp=title, abstract, subject headings, heading word, drug trade name, original title, device manufacturer, drug manufacturer name]

10. monitrice?.mp. [mp=title, abstract, subject headings, heading word, drug trade name, original title, device manufacturer, drug manufacturer name]

11. (peer adj (volunteer? or counsel$ or outreach or support)).mp. [mp=title, abstract, subject headings, heading word, drug trade name, original title, device manufacturer, drug manufacturer name]

12. "peer to peer".mp. [mp=title, abstract, subject headings, heading word, drug trade name, original title, device manufacturer, drug manufacturer name]

13. "mother to mother".mp. [mp=title, abstract, subject headings, heading word, drug trade name, original title, device manufacturer, drug manufacturer name]

14. "family to family".mp. [mp=title, abstract, subject headings, heading word, drug trade name, original title, device manufacturer, drug manufacturer name]

15. (church based adj3 (intervention$ or program$ or counsel$)).mp. [mp=title, abstract, subject headings, heading word, drug trade name, original title, device manufacturer, drug manufacturer name]

16. (linkworker? or link worker?).mp. [mp=title, abstract, subject headings, heading word, drug trade name, original title, device manufacturer, drug manufacturer name]

17. barefoot doctor?.mp. [mp=title, abstract, subject headings, heading word, drug trade name, original title, device manufacturer, drug manufacturer name]

18. (home adj (care or aid or aides or nursing or support or intervention? or treatment? or visit$)).mp. [mp=title, abstract, subject headings, heading word, drug trade name, original title, device manufacturer, drug manufacturer name]

19. ((care or aid or aides or nursing or support or intervention? or treatment? or visit$) adj3 (lay or volunteer? or voluntary)).mp. [mp=title, abstract, subject headings, heading word, drug trade name, original title, device manufacturer, drug manufacturer name]

20. 18 and 19

21. Lay Health Worker.mp. [mp=title, abstract, subject headings, heading word, drug trade name, original title, device manufacturer, drug manufacturer name]

22. "Letter [Publication Type]"/

23. "Editorial [Publication Type]"/

24. 22 or 23

25. exp Pneumonia/

26. pneumonia.mp. [mp=title, abstract, subject headings, heading word, drug trade name, original title, device manufacturer, drug manufacturer name]

27. exp Anti-Bacterial Agents/

28. antibiotic$.mp. [mp=title, abstract, subject headings, heading word, drug trade name, original title, device manufacturer, drug manufacturer name]

29. exp Child/

30. exp Infant/

31. (children or infant$ or pediatric or paediatric).mp. [mp=title, abstract, subject headings, heading word, drug trade name, original title, device manufacturer, drug manufacturer name]

32. or/29-31

33. malaria.mp. or exp Malaria/

34. exp Diarrhea/ or diarrhoea.mp.

35. "acute respiratory infection".mp. or exp Respiratory Tract Infection/

36. exp Lower Respiratory Tract Infection/ or "acute lower respiratory infection".mp.

37. ("Subsaharan Africa" or "Sub-Saharan Africa").mp. [mp=title, abstract, subject headings, heading word, drug trade name, original title, device manufacturer, drug manufacturer name]

38. (pneumonia in ti or pneumonia in ab).mp. [mp=title, abstract, subject headings, heading word, drug trade name, original title, device manufacturer, drug manufacturer name]

39. 25 or 26 or 27 or 28 or 33 or 34 or 35 or 36

40. exp "Africa South of the Sahara"/

41. 37 or 40

42. health auxiliary.mp. or exp Health Auxiliary/

43. voluntary worker.mp. or exp Voluntary Worker/

44. Home Care.mp. or exp Home Care/

45. community care.mp. or exp Community Care/

46. exp Peer Group/

47. exp CAREGIVER/

48. exp Social Support/

49. village health worker.mp.

50. 1 or 2 or 3 or 4 or 5 or 6 or 7 or 8 or 9 or 10 or 11 or 12 or 13 or 14 or 15 or 16 or 17 or 20 or 42 or 43 or 44 or 45 or 46 or 47 or 48 or 49

51. 21 or 50

52. 51 not 24

53. 32 and 39 and 41 and 52

54. limit 53 to yr="1987 - 2007"

Ovid key

(ADJ)The Adjacent operator retrieves records with search terms next to each other.

($) Unlimited truncation retrieves all possible suffix variations of the root word indicated.

(?)The wild card character ‘?’ is used within or at the end of a search term to substitute for one or no characters.

**CAB Direct**
(((Subsaharan Africa OR Sub-Saharan Africa OR Africa South of the Sahara) AND (((pneumonia OR acute respiratory infection) OR (acute lower respiratory infection) OR (malaria OR fever OR antimalarial*)) AND (ADDEDDATE >= 19870101))) OR ((sub-saharan africa OR subsaharan africa OR Africa South of the Sahara) AND (({5C, 0H, XH, 0J, XJ, 0L, XL, 0Y, 1T, X1, 2T, X2, X8, 0U, XN, ZS}) in SCCODES AND (((diarrhoea) OR (diarrhea) OR (transmissible gastroenteritis virus)) AND (ADDEDDATE >= 19870101))))) AND ((({5C, 0H, XH, 0J, XJ, 0L, XL, 0Y, 1T, X1, 2T, X2, X8, 0U, XN, ZS}) in SCCODES AND (((community health worke*) OR (community health aid*) OR (village health worke*) OR (lay health worke* OR community voluntee*)) AND (ADDEDDATE >= 19870101))) OR ((Subsaharan Africa OR Sub-Saharan Africa OR Africa South of the Sahara) AND (((community based distributo*) OR (mother coordinato*) OR (traditional birth attendan*) OR (patent medicine deale*)) AND (ADDEDDATE >= 19870101))))

CAB Direct Key

Index of Codes are listed at:

http://217.154.120.6/CABDIRECT/_CABHLP/CABDIRECT/Fields.htm
