# Supplementary material for: Thirty years after Alma-Ata: a systematic review of the impact of community health workers delivering curative interventions against malaria, pneumonia and diarrhoea on child mortality and morbidity in sub-Saharan Africa
Source: Hum Resour Health. 2011 Oct 24;9:27. doi: 10.1186/1478-4491-9-27 (PMC3214180; doi:10.1186/1478-4491-9-27)
Supplement: Additional file 2 — Data extraction sheet [file 1478-4491-9-27-S2.DOCX]

## Additional file 2 - Data extraction sheet

1. **OUTCOME ACTION ?**

**2) Strength of evidence**

1. **Study No.**
2. **Title**
3. **Author/Year/Journal:**
4. **Setting**
5. **Design**

| **Geographic setting**  **(please tick)**  **?Road access** | | **Rural** | **Formal**  **Urban** | **Informal Urban**  **Settlement** |
| --- | --- | --- | --- | --- |
| **Health care setting for CHW intervention** | **Home** | | **Primary care facility** | **Other**  **(please specify)** |
| **Country** | | | | |

1. **Typology of Context**

| **Burden of Disease**  **(words & no.s)** | **Malaria** | **Diarrhoea** | **ARI** |
| --- | --- | --- | --- |

| **Functioning of Prim. Care Level Health care** |  |
| --- | --- |
| **Decentralisation of Health Service Control** |  |
| **Employment Alternatives for CHWs** |  |

1. **CHW Programme Overview**

| **How old is it ?** |  |
| --- | --- |
| **Set up & overseen by who ?**  **(i.e nat. programme or special eg NGO)** |  |
| **No. of CHWs in Programme** |  |
| **No. of total programme beneficiaries** |  |
| **Attrition Rate of CHWs** |  |

**7) CHW Role & Selection**

| **Extract the term**  **used to describe the**  **community health worker**  **in the text (e.g.**  **traditional birth attendant)** |  |
| --- | --- |

**Tasks performed**

| **Describe the health care tasks performed with consumers (patients, groups v individ, drugs, freq. of visit, setting, activites to increase compliance**  **+ all aspects of intervention.** |  |
| --- | --- |
| **Curative v Preventive** |  |
| **Weekly pattern of activity** |  |

**Selection criteria for CHWs (e.g. demographics, selected by who- ? villagers, etc)**

|  |
| --- |

**8)CHW Characteristics**

| **Education** |  | |
| --- | --- | --- |
| **Sex mix** |  | |
| **Age** |  | |
| **Ethnicity & Religious Representation** |  | |
| **Paid ?**  **(money or in kind)** |  | **If Y, by whom ?** |

**9) CHW Training**

| **Duration**  **Methods Used** |  |
| --- | --- |
| **Content (eg. curative, prevention, teaching + group facilitation skills, record keeping)** |  |
| **By Whom (eg. Villagers/gov/ Prim care worker)** |  |
| **Educational Materials Provided** |  |
| **Refresher Training**  **How often, how long, by whom ?** |  |

**10) CHW Supervision/Support/Management**

| **Who supervises ?**  **(eg village/PHC worker/gov)** |  |
| --- | --- |
| **How ? (eg what data/feedback)** |  |

**Incentives**

| **? Opportunity for promotion** |  |
| --- | --- |
| **Profit-making opportunities ?** |  |

**11) Study Participants- the consumers**

| **Health Problems managed/treatments**  **Received/education given** |  |
| --- | --- |
| **Age, demography** |  |
| **Sex** |  |
| **Brief description of target group eg. indication of Social economic status** |  |
| **Cultural background** |  |

**12) RCTs Methodological Quality & Results**

|  | **Indicate if done, not done or unclear** | **Give details** |
| --- | --- | --- |
| **Concealment of Allocation** |  |  |
| **Follow Up of CHW** |  |  |
| **Follow Up of consumers** |  |  |
| **Blinded assessment of primary outcome?** |  |  |
| **Baseline measurement** |  |  |
| **Reliable Primary Outcome measure** |  | **(?when measured)** |
| **Protection against contamination**  **(eg. From intervention Group to Control Group)** |  |  |
| **Intention to treat**  **analysis?** |  |  |

**CHWs or Groups of CHWs**

| **Unit of Allocation/**  **Randomization** | **Individual patient** | **Practice/setting** |
| --- | --- | --- |
|  |  |  |
| **Unit of intervention** |  |  |
| **Potential for unit of analysis error for some outcomes** |  | |
| **If yes, is the problem acknowledged?** |  | |
| **If yes, is there any**  **adjustment/correction** |  | |

**CHWs (if study begins at programme onset)**

|  | **No.**  **Approached** | **No. allocated**  **to groups** | **No. trained** | **No. and ( %)**  **followed up** |
| --- | --- | --- | --- | --- |
| **Overall** |  |  |  |  |
| **Intervention 1 (CHW)** |  |  |  |  |
| **Intervention 2** |  |  |  |  |
| **Control** |  |  |  |  |

**Are health activities of control group clearly the same apart from the intervention ? (Y/N/U)**

|  | **Details** |
| --- | --- |

**Consumers (patients):**

|  | **No. at baseline who agreed to participate** | **No. at baseline who eligible to participate** | **No. received intervention** | **No. and % followed up** |
| --- | --- | --- | --- | --- |
| **Intervention** |  |  |  |  |
| **Control** |  |  |  |  |

**Primary Outcome Results**

| **Primary Outcome**  **Measures** | **Method of measurement** | **Control** | **Intervention 1** | **Statistical**  **Significance**  **(P+C1)** |
| --- | --- | --- | --- | --- |
|  |  |  |  |  |
| **Any adverse effects resulting from the intervention ?** |  |  |  |  |
| **Other outcomes ?** |  |  |  |  |

**13) References:**

| **References to be included in the review or followed up e.g. references that describe the intervention; measures used; further results; possible other trials** |  |
| --- | --- |

**14) Narrative summary of the impact of the intervention on the primary outcome:**

|  |
| --- |

**Notes:**

**12) Non-Randomised Studies: Methodological Quality & Results**

**Was SELECTION BIAS present with respect to:**

|  | **Y/N/Unclear** | **Details** |
| --- | --- | --- |
| **Sampling?** |  |  |
| **Recruitment?** |  |  |
| **Eligibility?** |  |  |
| **Unit & Method of assignment?** |  |  |

**Were control and intervention entrants explicitly assessed for similarity ?**

**(Y/N/Unclear) (Details)**

|  |  |
| --- | --- |

**With respect to the following factors is this similarity clear ?**

**(Y/N/Unclear) (Details)**

| **Severity of presentation** |  |  |
| --- | --- | --- |
| **Age** |  |  |
| **Sex** |  |  |

**Were baseline comparisons of those lost to follow-up and those retained performed?**

**(Y/N/Unclear) (Details)**

|  |  |
| --- | --- |

**Was study population at baseline similar to population of interest ?**

**(Y/N/Unclear) (Details)**

|  |  |
| --- | --- |

**CONFOUNDING**

**Evidence that the following health preventative/curative/damaging factors were objectively assessed (YES/NO/UNCLEAR & DETAILS):**

| **Public health care** |  |  | |
| --- | --- | --- | --- |
| **NGO/mission** |  |  | |
| **Traditional Healers** |  |  | |
| **TBAs** |  |  | |
| **Private health care** |  |  | |
| **Drug sellers** |  |  | |
| **Economic factors** |  |  | |
| **Geographical factors (eg roads, access to healthcare)** |  |  | |
| **Environmental factors (eg rains, famine)** |  |  | |
| **Secular trends** |  | |  |
| **Other** |  | |  |

**Were appropriate statistical methods used to control for confounding ?(YES/NO/UNCLEAR & DETAILS)**

|  |  |
| --- | --- |

|  | **Indicate if done/not done/unclear** | **Give details** |
| --- | --- | --- |
| **Follow Up of CHW** |  |  |
| **Follow Up of consumers** |  |  |
| **Blinded assessment of primary outcome?** |  |  |
| **Reliable Primary Outcome measure?** |  | **(? When measured)** |
| **Protection against contamination**  **(eg. From intervention Group to Control Group)** |  |  |
| **Intention to treat**  **analysis?** |  |  |

**CHWs or Groups of CHWs**

| **Unit of Allocation** | **Individual patient** | **Practice/setting** |
| --- | --- | --- |
|  |  |  |
| **Unit of intervention** |  |  |
| **Potential for unit of analysis error for some outcomes** |  | |
| **If yes, is the problem acknowledged?** |  | |
| **If yes, is there any**  **adjustment/correction** |  | |

**CHWs (if study begins at programme onset – Y / N ?)**

|  | **No.**  **Approached** | **No. allocated**  **to groups** | **No. trained** | **No. and ( %)**  **followed up** |
| --- | --- | --- | --- | --- |
| **Overall** |  |  |  |  |
| **Intervention 1 (CHW)** |  |  |  |  |
| **Control** |  |  |  |  |

**Consumers (patients):**

|  | **No. at baseline who agreed to participate** | **No. at baseline who eligible to participate** | **No. received intervention** | **No. and % followed up (? >80%)** |
| --- | --- | --- | --- | --- |
| **Intervention 1** |  |  |  |  |
| **Control** |  |  |  |  |

**Primary Outcome Results**

| **Primary Outcome**  **Measures** | **Method of measurement** | **Control** | **Intervention 1** | **Statistical**  **Significance**  **(P+C1)** |
| --- | --- | --- | --- | --- |
|  |  |  |  |  |
| **Any adverse effects resulting from the intervention ?** |  |  |  |  |
| **Other outcomes ?** |  |  |  |  |

**13) References:**

| **References to be included in the review or follow up**  (e.g. references that describe the intervention; measures used; further results; possible other trials) |  |
| --- | --- |

**14) Narrative summary of the impact of the intervention on the primary outcome:**

|  |
| --- |

**Notes + strength of evidence summary statement.**
